# Shanna H Swan: environmental exposure to chemicals and their consequences for human fertility

**DOI:** 10.2471/BLT.25.030425

**Published:** 2025-04-01

## Abstract

Shanna H Swan talks to Gary Humphreys about the impact on human health of exposures to endocrine-disrupting chemicals in the environment.

*Q: Your bachelor’s degree was in mathematics and logic. How did you come to focus on biostatistics?*

A: I was just fascinated by the topic, but also very lucky with my teachers. I studied under the Polish biostatistician Agnes Berger for my master’s at Columbia. Then at Berkeley, another icon, Jerzy Neyman, took me into the department and guided my training. In terms of my professional interest, that really came into focus when I went to work at Kaiser Permanente Division of Research in 1976. I was tasked with looking at health outcomes in oral contraceptive users, and published multiple papers exploring what I later recognized to be early examples of endocrine disruption (the disruption of hormone production, secretion, transport, action or elimination). That was also where I first applied the important lessons that I had learned during my biostatistics training at Columbia regarding the importance of familiarizing oneself with the processes driving or informing data generation and collection.

*Q: Why is that so important?*

A: Because those processes can change the results you end up with. For example, I remember watching a colleague discarding outlier results as a way of ‘tidying up’ the data – a practice I later helped to correct. This investigative, under-the-hood approach served me throughout my career, including when I decided to study sperm counts and immersed myself in the lab work to understand sperm counting procedures firsthand, enhancing my grasp of data quality and variability. I think this is really important, rather just taking data at face value.

*Q: When did you develop your interest in the population-level health impact of environmental exposures?*

A: It was during my time at the California Department of Public Health in the mid-1980s. The department received a request from a health officer in Santa Clara County, who reported a potential link between semiconductor plant leaks and increased miscarriage rates. I was asked to lead the investigation, and it was a transformational experience for me. I attended community meetings, visited the industry stakeholder, and even investigated the leak itself which was discovered by a worker who noticed a problem with a storage tank overflow system. My research revealed that women residing near those plants experienced miscarriage rates far higher than those in other parts of the county. This incident led to a series of studies on the reproductive effects of environmental exposures, which I organized under a newly formed reproductive epidemiology programme in the health department.

*Q: You are best known for your work on the impact of exposure to environmental risks on human sperm count. When did that work start?*

A: In 1995, when I was invited to join a committee of the National Academy of Sciences. The committee was exploring hormonally active agents in the environment, and I was tasked with reviewing a Danish study, claiming to show a 50% drop in sperm counts between 1938 and 1991. The committee wasn’t convinced, and I admit that had doubts myself. But when I looked at the original 61 studies, and after adjusting for a number of possible confounding factors, I found the results to be unchanged. I then looked at 40 more studies, published after the initial Danish meta-analysis, and saw that the results were again confirmed. It was astonishing to me, and I decided to take a look at the possible causes of the decline – something that had not been addressed in the Danish study. So, I went to my boss at the California Department of Health Services with a proposal and he said, and I quote, “This does not get my engine going.” Fortunately, I received an invitation from Fred vom Saal, one of the scientists on the committee who had a particular interest in endocrine disruption, to join him at the University of Missouri. He said, “Come to Missouri and we can make trouble together.” And we did.

*Q: In what way?*

A: I initiated a study in September 1999, enrolling roughly 900 expectant parents in four US locations. It was called the Study for Future Families and revealed significant variations in sperm quality between locations, such as Columbia, Missouri and Minneapolis. For example, male participants in a semi-rural Missouri area had half as many moving sperm as men in urban Minneapolis. Thinking that the use of pesticides in agriculture might have something to do with it, I sent the Missouri men’s urine samples (which I’d collected at the same time as I collected their semen) to the Centers for Disease Control and Prevention (CDC). The samples with higher levels of eight pesticides relative to their counterparts in urban Minneapolis were from the participants with the poorer semen quality.

*Q: When did you become interested in exposures to the chemicals present in plastics?*

A: It was around the same time, and it was sparked by a conversation I had on a flight to Japan for a conference in the early 2000s. I sat next to John Brock, a chemist from the CDC, and he started talking to me about phthalates, a group of chemicals used to enhance the flexibility and durability of plastics. They are now understood to be potent endocrine disruptors, but back in the early 2000s their impact was just beginning to be understood. John explained that the National Toxicology Program had identified a cluster of adverse outcomes linked to phthalates referred to as the phthalate syndrome, which involved the under-development of the male genitalia. In rodents this underdevelopment was measured (among other markers) in terms of the distance between the anus and the genitals (anogenital distance or AGD), a marker of reproductive development which is influenced by testosterone levels. I initiated a study to see if the phthalate syndrome was also observable in humans. Using the urine from mothers in the Study for Future Families, and with John’s help at the CDC, I was able to determine the mothers' phthalate levels when pregnant. Then I compared those levels with AGD and other markers of the phthalate syndrome such as penile size and testicular descent in their male offspring. I found that women in the upper quartile of exposure to several phthalates were significantly more likely to have a son with a shorter-than-average AGD than those in the lowest quartile.

*Q: How were you able to establish the association between a shorter AGD and lower sperm count?*

A: We conducted a study of college students, in Rochester New York in 2009 which confirmed our hypothesis. The finding was subsequently supported by a study done by Michael Eisenberg at Stanford in 2011. To further confirm our findings, we initiated the Infant Development and Environment Study now known as TIDES. This time, we collected urine samples from the mothers during each trimester to pinpoint the critical windows of exposure. When we examined the newborns shortly after birth, we found, once again, that a mother’s phthalate exposure was related to her son’s genital development.

“In four decades sperm counts more than halved.”

*Q: Were substances other than phthalates considered in your research?*

A: Indeed, the stored urine samples have been analysed for other chemicals, including bisphenol A, which is used in plastics and also acts as an endocrine disruptor. We have also done more work on pesticides. I’d also like to draw attention to our sperm decline meta-analyses, published first in 2017 and later updated in 2022. The analyses revisited the question of sperm count decline that was first raised by Dr Elisabeth Carlsen in the 1992 Danish study. In 2017, we examined a total of 185 studies, using meta-analysis methods not available in the early 90s. That meta-analysis revealed a 52% decline in sperm concentration and a 59% decrease in total sperm count among men from North America, Europe, Australia and New Zealand between 1973 and 2011. In four decades, sperm count in those countries more than halved. When that study was published in 2017, it made big news and eventually led to me writing *Countdown*.

*Q: What prompted you to write a book for a general audience?*

A: It was partly to get the message out as widely as possible, but also to reflect women's perspectives more fully, since our initial findings were more male-focused. While we didn't analyze women's data specifically for the book, my background in women's reproductive health enriched our approach, emphasizing the intersection of male and female reproductive health issues. I've dedicated much of my career to women's reproductive health, going right back to the work I did on semiconductors.

*Q: The global plastics treaty negotiations stalled in 2024, with delegates failing to reach a consensus on key issues, including capping plastic production and managing hazardous chemicals in plastics. How can we move forward on this?*

A: Addressing this challenge is going to require global cooperation, focused on finding alternatives to the harmful chemicals used in plastic and reducing plastic production and use. Unfortunately, the challenges faced have been compounded by the politicization of the topic. My approach has been to focus on the science and the direct impact of these environmental factors on health, avoiding the political fray by concentrating on educating the public and demonstrating practical solutions through specific interventions such as substituting everyday items like storage containers, cleaning products, and even shower curtains with safer alternatives. We're also looking at the effects of substituting plastic containers for glass in the collection and growth of embryos and semen samples. Preliminary findings suggest that removing plastics can lead to better outcomes in reproductive technologies. The response to these projects has been overwhelmingly positive.

*Q: Despite the challenges faced in reducing plastic manufacture and use, you sound quite positive about the prospects for change. What inspires that optimism?*

A: I strongly believe in the power of informed action and see proof of it on a regular basis. For instance, during a recent retreat, a tech entrepreneur was so intrigued by our discussion on toxins in food that he launched a project to test food for plastic content. This initiative has sparked widespread interest and dialogue about environmental health. So, yes, I’m optimistic and I’m excited to see how our initiatives will continue to evolve and the impact they’ll have on public health and environmental policies.

